# Influence of polymerisation on the reversibility of low-energy proton exchange reactions by Para-Aminothiolphenol

**DOI:** 10.1038/s41598-017-13589-5

**Published:** 2017-11-13

**Authors:** Divya Balakrishnan, Guillaume Lamblin, Jean Sebastien Thomann, Jerome Guillot, David Duday, Albert van den Berg, Wouter Olthuis, César Pascual-García

**Affiliations:** 1 Luxembourg Institute of Science and Technology (LIST), 41 Rue du Brill, L-4422 Belvaux, Luxembourg; 2 BIOS-Lab on a chip group, MESA+ Institute of Nanotechnology, MIRA Institute for Biomedical Technology and Technical Medicine, University of Twente, 7500 AE Enschede, The Netherlands

## Abstract

The reversibility of redox processes is an important function for sensing and molecular electronic devices such as pH reporters or molecular switches. Here we report the electrochemical behaviour and redox reversibility of para-aminothiolphenol (PATP) after different polymerisation methods. We used electrochemical and photo-polymerisation in neutral buffers and plasma polymerisation in air to induce reversible redox states. The chemical stoichiometry and surface coverage of PATP in the polymerized layers were characterized by X-ray photoelectron spectroscopy (XPS), while cyclic voltammetry (CV) was used to measure the charge transfer, double layer capacitance and electrochemical rate of the layers during successive potential cycles. Our results show that the surface coverage of the redox active species is higher on electro-polymerised samples, however, after consecutive cycles all the methods converge to the same charge transfer, while the plasma polymerised samples achieve higher efficiency per molecule and UV polymerised samples have a higher electron transfer rate.

## Introduction

Surfaces with active reversible redox states are key elements in the fabrication of sensors, batteries, supercapacitors or chemical switches^1^. Organic materials using proton exchange reactions, have an additional interest due to the promise of a greener approach and better efficiencies than alkaline metals^2,3^. Aminothiolphenol (ATP) is one simple molecule that is able to form self-assembled monolayers (SAMs) on noble metals, which can be used to modify the physico-chemical surface properties or to initiate the growth of well-ordered molecular layers, maintaining a good electrical conductivity. The mercapto group and benzene ring of ATP act as the linker and separator respectively. The amino group can exchange protons with the electrolyte or be used as a target for chemical bonds. In the para aminothiolphenol (PATP) the mercapto and amino groups are opposite to each other providing higher conductivity, due to the more extensive electronic conjugation with respect to other isomeric states^4^. Polymerisation of PATP adsorbed layers on noble metals leads to different reversible redox states corresponding to different molecular coupling of two PATP molecules. The redox reactions occur at low potential, between 0 and 200 mV, and involve the exchange of two protons and two electrons. These polymerised forms of PATP have an important role for many of its applications because they support stable states with redox reversibility in contrast to the monomeric state^5^.

PATP has been used for the design of chemical sensors and switches or synthesis of conductive composites in combination with metallic nanoparticles and carbon materials^6^. PATP is one of the most important pH molecular reporters thanks to the interplay of the amine group with protons^7^. In acidic solution, PATP retains its aromatic state and in alkaline or neutral solution, it dimerizes into quinonoidic state 4,4′- dimercaptobenzene (DMAB). When chemisorbed to nanoparticles using SERS measurements the intensity of double bonds between nitrogen molecules in DMAB are detected, reporting pH between 3 and 7 in miniaturized environments^6,7^. The modification of the pH by PATP would be the counterpart concept of this application by inducing proton release using an applied current. The pH variation by this process is limited by the reduced surface area of the electrodes compared to the total volume in solution. Using a nanocomposite coating it was possible to change the pH in a small volume and reversibly activate, a pH dependent DNAzyme^8^ which opens the door to new applications like the control of the chemical environment by electronics or DNA computation^9,10^. One of the drawbacks of the electrochemical generation of protons is its limited reversibility.

The photo-detection and electro-production of protons are almost equivalent processes that show very different reversibility. The susceptibility to electrical degradation of the PATP layers depends on the polymerised state induced. In literature, two main routes of polymerisation of PATP have been described to different extents: electro-polymerisation (e-poly) and photo-polymerisation. E-poly layers are achieved by applying a potential on the PATP SAMs. Hayes and Shannon reported the first study of the e-poly of PATP in acidic solution and explained the oxidation of PATP to form 2-(4-mercaptophenylamino) benzoquinone which is bound to the surface through the sulphur, resulting in a head to tail polymerisation^11^ confirmed by other authors^4,12,13^. Photo-polymerisation is induced by illuminating UV light on the PATP SAMs. Different studies suggested that UV-polymerisation (UV-poly) of PATP favours head to head polymerisation and results in different redox active species (DMAB) similar to those described in pH sensors^14–16^. Polymerised states can also be achieved by plasma treatment: direct energy transfer of plasma can produce damages, but plasma can also create intermediate species (radicals, ions, electrons, photons) that permit the polymerisation of PATP. Plasma treatments of aniline at low pressure, in presence of *O*_2_ are known to lead to polymerisation, etching or oxidation depending on the plasma parameters as discussed in other papers^17,18^. Recent works have shown that plasma polymerisation can also be a dominant process at atmospheric pressure by choosing the proper electrode configuration^19^ or the electrical signal^20,21^. Polyaniline nanofibers or nanoparticles were recently obtained with an atmospheric pressure plasma process^19^.

Regarding the importance of reversibility of the redox states in polymerised layers, to the best of our knowledge, no comparison of the efficiency of the redox states has been reported. In this paper we report the reversibility of polymerised states of PATP induced by electrochemical, UV and plasma methods. Although plasma-polymerisation has been used for polyaniline, plasma had never been used to treat aniline-like SAMs, even if plasma polymerisation holds advantages in the treatment of large samples. Our main goal is to investigate the efficiency of the polymerisation processes to provide reversible redox states. X-ray photoelectron spectroscopy is used to characterize the chemical configuration of the polymerised states and the surface coverage, while the efficiency of redox reactions was accessed using CV to measure the charge transfer (CT), film capacitance and reaction rates.

## Results and Discussions

### Polymerisation of PATP layers

**Figure 1:**
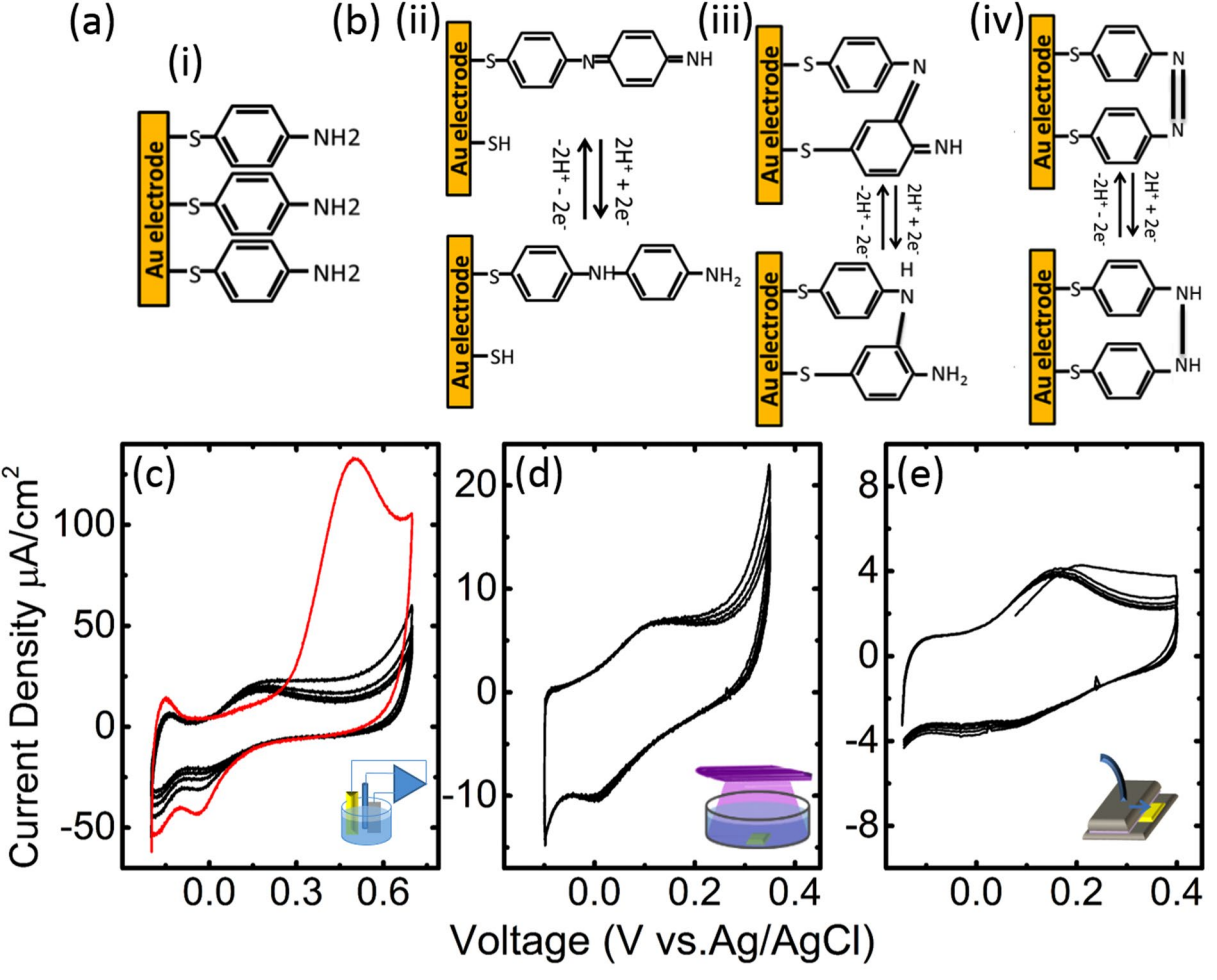
Schematic representation of the PATP monolayers adsorbed on gold (**a**) and most common products of polymerisation (**b**) (in a structural formula representation). Representative cyclic voltammograms corresponding to the electropolymerisation of PATP(**c**) with the first and subsequent cycles (in red and black respectively) and UV (**d**) and plasma polymerised (**e**) samples respectively.

Figure 1 shows a representation of the PATP chemisorbed layers (Fig. 1(a)) along with the three main reactions generally recognised in reversible proton exchange^5^ (Fig. 1(b)). Figure 1(c) shows one representative cyclic voltammogram corresponding to the electropolymerisation of one of the PATP modified Au electrodes. The current was acquired driving the potential between −0.2 and 0.7 V against a Ag/AgCl reference electrode for 6 cycles with a rate (ʋ) of 100 mV/s. In cycle 1 (in red) a large irreversible oxidative wave is observed at 0.5 V with a full width half maximum (FWHM) of the peak of 0.2 V. According to literature this peak can be attributed to the oxidation of PATP and the consecutive polymerisation that occurs when an oxidised molecule couples to an adjacent one to yield a head to tail dimer (Fig. 1(b–ii)) ^11^. An oxidation-reduction couple at 0.17 and −0.03 V with respective FWHMs of 0.2 and 0.1 V respectively were observed after cycle 2 (in black) and attains a steady state within the subsequent cycles. According to literature, these peaks are attributed to the oxidation and reduction of 4-mercapto-N-phenylquinone diimine and 4-mercapto-aminodiphenylamine respectively, which involve the exchange of two protons and two electrons. Below 0.1 V an increase of the negative current that decreases on successive cycles is observed. In literature was attribured to the reduction of thiols and to side reactions^11,12^. UV polymerised samples were obtained by illuminating the functionalised samples with a 365 nm lamp under 0.1 M phosphate buffer. Figure 1(d) shows a representative cyclic voltammogram from one of the UV-polymerised samples. The CV shows the first 5 cycles (*ʋ* = 0.1 V/s). The potential range was narrowed between −0.1 and 0.4 V to avoid further electropolymerisation, although a small rise of the current above 0.3 V can be attributed to electropolymerisation of unpolymerised molecules. The quasi-reversible oxidation and reduction peaks corresponding to the proton exchange reactions of the amine groups were observed at 0.1 and 0.06 V respectively. While the UV polymerisation has not been studied in literature as much as electropolymerisation, several papers suggest that aniline of thiol aniline in neutral solution undergo head to head polymerisations as schematically shown in Fig. 1(b)-(iii–iv) also exchanging two protons and electrons per reaction^15^. Plasma samples were obtained using an open plasma reactor with a direct plane-to-plane configuration^22^. Figure 1(e) shows the first five cycles of a representative cyclic voltammogram. The reversible redox peaks corresponding to the amine oxidation/reduction were observed at 0.16 and 0.08 V respectively. Also a small contribution of electropolymerisation was observed on the first cycles. The most probable polymerisation reactions during the plasma treatments are *H* abstraction on the benzene rings leading to a *C-C* bonding between benzene rings from adjacent PATP molecules as proposed in^18^, or *H* abstraction on the *NH*_2_ groups leading to *N* = *N* bonds between PATP adjacent molecules. Plasma can also lead to oxidation of benzene rings (*-OH* addition) or *NH*_2_ groups and to some thiol or *C-NH*_2_ bond breaking leading to a partial etching of the SAM. Here the conditions were optimized to favour the plasma polymerisation processes instead of plasma oxidation and plasma etching processes by exploring the plasma conditions which provided the greater CT for redox reactions.

### XPS analysis

**Figure 2:**
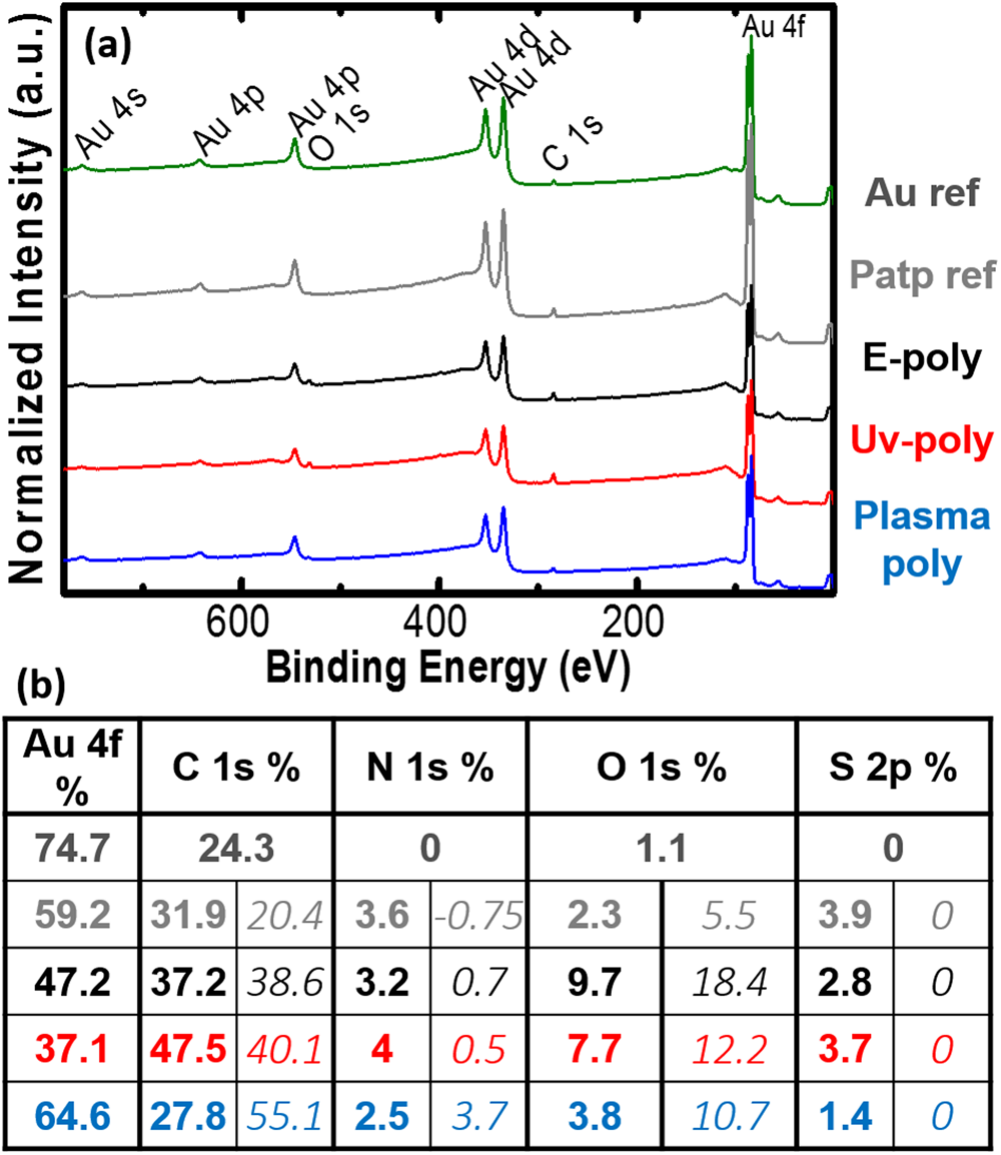
(**a**) XPS survey spectra from Plasma, UV, e-poly samples (blue, red and black respectively) and the PATP and Au samples (grey and green respectively). (**b**) Elemental composition (in atomic %) for each sample (bold) and percent of excess of each element with respect to the PAPT stoichiometry considering *S* content as reference (italic numbers).

XPS can provide insights of the chemical composition of thin layers. Figure 2(a) shows the survey spectra for the PATP and Au reference samples, as well as electro, UV and Plasma polymerised samples (grey, green, black, red and blue respectively, colour convention is maintained herein). The table shows the extracted elemental composition in atomic percentage (in bold). The Au reference samples exhibit *C* and *O* contamination (24 and 1% respectively) due to exposure to air. The reference unpolymerised PATP sample exhibited also *N* and *S* present in PATP molecule. In order to study the extrinsic chemical elements in the functionalised layers, we separated the Au contribution originated in the substrate, and calculated the theoretical percentage for each element using the *S* contribution as a reference and the PATP stoichiometry. The excess is reported in the table in Fig. 2 in italic numbers. Thus 20% of the *C* detected in the PATP reference sample is attributed to contamination, which is similar to the amount found in the Au reference. In addition some extra presence of *O* (5%) is attributed to oxidation of PATP. The e-poly samples showed an increase in the *C* and *O* in excess (39% and 18% respectively). This increase of contaminants and in particular the high increase of oxygen can be attributed to the head to tail polymerisation characteristic of electropolymerisation, which leaves unprotected thiols that may oxidise and attract carbon to the surface when exposed to air. In UV-poly samples there is a comparative decrease of *O* respect to e-poly samples even if remains significantly higher than in the reference sample (10% of the total content), probably due to the contribution of oxidative species formed in solution during UV-poly. The plasma-poly samples, exhibited a decrease of the organic content and an increase of the signal from the Au substrate, the highest *C* excess (55.1%), a small increase of the *N* signal as well as a moderate oxidation respect to the PATP reference. Our interpretation is that plasma-poly samples suffered some etching during the plasma treatment that could lead to an increase of the contamination since the uncovered Au surface has a very high surface energy and oxidation of the layers due to the radicals formed in the plasma.

**Figure 3:**
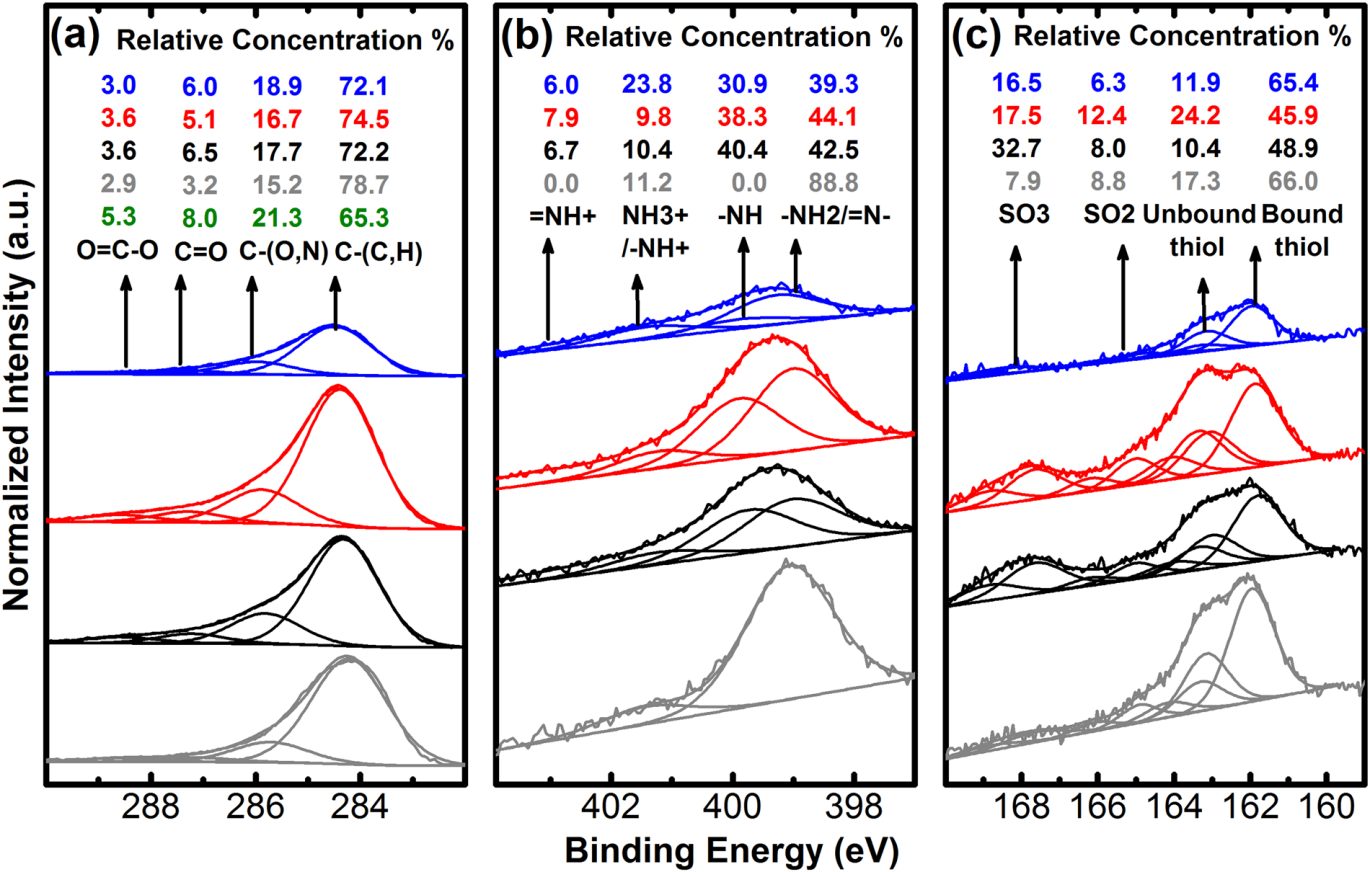
Core level spectra of the XPS analyses for the C, N and S contributions of the polymerised and PATP samples.

The fine structure of XPS peaks can also provide insights to the chemical bonds to clarify the nature of the adsorbed and polymerised layers. Figure 3 shows the narrow scans for *C 1* *s*, *N 1* *s* and *S 2p* of the polymerised and PATP reference samples, including the peak analysis of the fine structure of the bonds. The *C 1* *s* core level spectrum (Fig. 3(a)), shows four peaks that have been assigned as follows: *C–*(*C*, *H*) at 284.5 eV, which is the main contribution as expected from aromatic compounds, *C–*(*O*, *N*) located at 286 eV, *C* = *O* at binding energy 287.6 eV and *O* = *C–O* centred on 288.8 eV. As expected, the four samples have very similar spectra as the C bonds are the same.

The study of the *N 1* *s* narrow scans (Fig. 3(b)) indicated the effectiveness of the polymerisation processes. The spectrum from the PATP reference presents only two contributions at 399.0 eV (~90% of the total nitrogen contribution) corresponding to primary amino groups (−*NH2*) and at 401.4 eV belonging to protonated or hydrogen-bonded amino groups (*NH3* + , *H* ^*…*^ *NH2*)^23–25^. It was already shown that amino groups are the dominating species for aromatic molecules whereas it can be the opposite for aliphatic amines^23,24^. Instead, polymerised samples presented four contributions, previously observed on polyaniline films^26–29^. The main line at 399.0 eV is characteristic of quinoid di-imine nitrogen groups (*-N* = ). The second main component at 399.8 eV is ascribed to benzenoid diamide nitrogen compounds (*-NH-*). The peak at 401.2 eV can be attributed to positively charged nitrogen species such as protonated amine (*-NH* + *-*) or to the oxidised amine (-*NO*). The contribution at 402.9 eV is attributed to the oxidised amine (*-NH* +  = ). The main signature of the polymerisation is the splitting of the primary amine groups into the quinoid di-imine and the benzenoid diamide groups.

**Figure 4:**
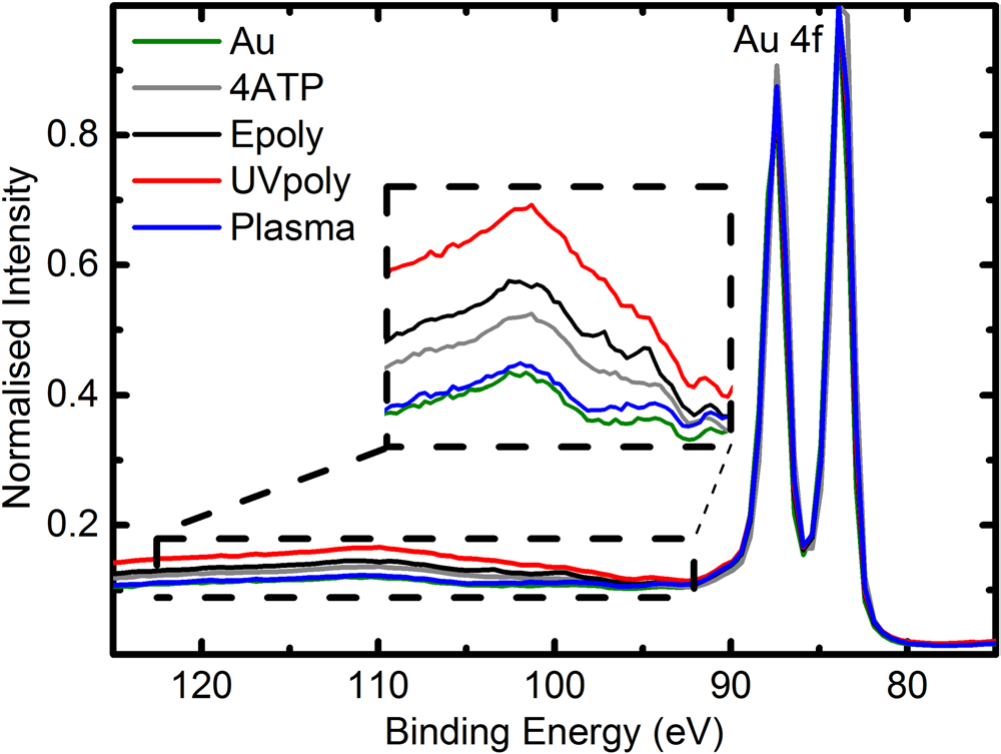
XPS inelastic scattering normalised by the *Au4f* signal of the polymerised and reference samples.

**Figure 5:**
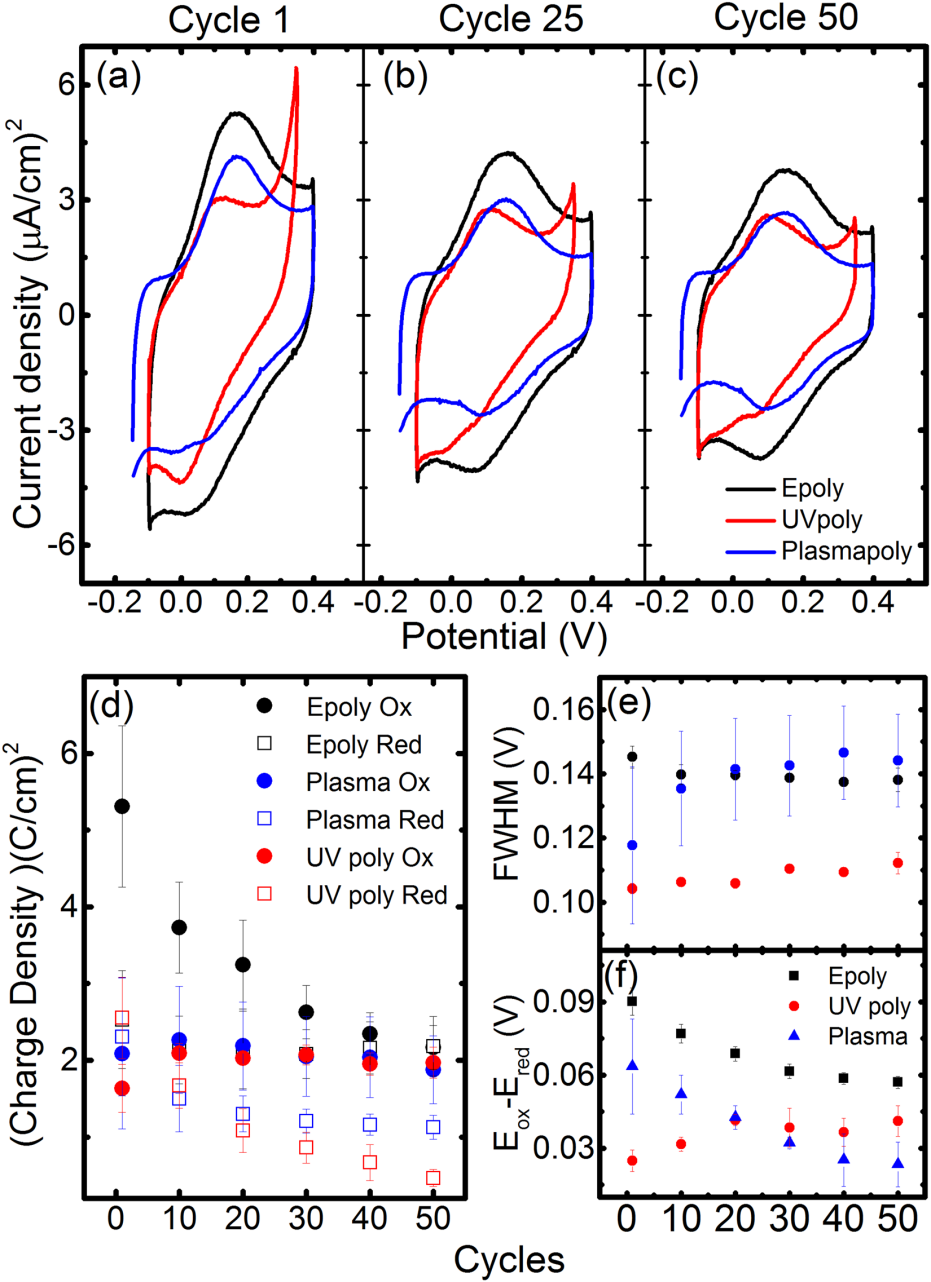
(**a**–**c**) CV after e- (black), UV (red)- and Plasma (blue) polymerisation methods for the cycles 2, 25 and 50 after polymerisation (first cycle was avoid to discard open circuit and unbalance of chemical equilibrium effects) (**d**) Charge transfer for the different polymerisation and for the oxidative and reduction peaks as a function of cycles. (**e**) FWHM of the oxidation peaks as a function of cycles, (**f**) Overpotential between oxidative and reduction peaks as the function of cycles.

Figure 3(c) shows the *S* 2*p* spectra. The line at 161.9 eV is attributed to thiol or thiolate strongly bonded to the gold surface^23,30–33^. The second doublet centred at 163.1 eV was previously ascribed to unbounded or non-chemisorbed thiol, thiolate or disulphide for high coverage^30,32,33^. Two additional components are detected at 165.0 and 167.6 eV, attributed to the presence of oxidised sulphur species *SO*_*2*_− and *SO*_3_− (sulfinate and sulfonate) respectively^23,30,33^. Most of the *S* signal from the reference PATP sample is associated with bound thiol. The component associated to the line at 163.1 eV may be due to excess of PATP adsorbed on top of the first monolayer. An increase of the unbounded thiol or thiolate contribution was observed in this batch of UV polymerised samples. We assigned it to the presence of more than one monolayer, which is in agreement with further data in the article (Figs 4 and 5). The peak at 168.5 eV is observed only for the polymerized species and we attribute it to the oxidized sulphur species originated in the unbound *–SH* produced also by head to tail polymerisation. The higher content of oxidised thiols in the case of e-poly is consistent with the interpretation of head to tail polymerisation as main component in this method.

The background due to inelastic scattering of photoelectrons can provide information about the layer thickness. Figure 4 shows the XPS inelastic electron scattering spectra from the polymerised and reference samples normalised to the *Au 4* *f* zero loss peak. The inset shows a blow up of the background displaying a ranking of *Au* substrate, plasma poly, unpolymerised PATP, e-poly and UV-poly from thinner layer to thicker layer. The signal in *Au* can be attributed entirely to contamination. Plasma-poly layers are thinner than the original PATP, which indicates a reduction of the layer coverage attributed to the etching effect of the plasma. The resulting larger layer thickness of the e-poly samples observed with respect to the reference PATP layers can be attributed to absorption of contaminants by the isolated thiols resulting of the head to tail polymerisation. Finally the thicker layer of PATP signals the presence of unbound thiols (Fig. 3(c)), which is consistent with the electrochemical data here after. This effect was particular of this batch of UV polymerised samples used for XPS and CV.

### Cyclic voltammetry evolution

The reversibility of the redox reactions, attributed to the proton exchange reactions of the polymerised PATP was studied with CVs for over 50 cycles (Fig. 5). e-poly, UV and plasma polymerised samples are presented with black, red and blue colours respectively. Figure 5(a–c) show the comparison of the evolution of the cyclic voltammograms for representative curves from one of the samples for each polymerisation at cycles 2, 25 and 50. The CVs are dominated by redox peaks at ~0.15 and ~0.08 V and according to the literature discussed above, in the case of electro and UV-polymerised samples are due to the reactions presented schematically shown in Fig. 1(b). e-poly samples displayed higher oxidative and background current densities than the others but decreased until cycle 50 where the redox currents of the three samples tend to converge. UV-poly samples exhibited lower redox currents than the e-poly and a considerable background current. An extra reduction peak around 0 V was observed in particular during the first cycles, which did not appear in other sets of UV-poly samples. We attribute this peak to the effect of the unbound PATP that we observed in some samples. The oxidative peaks displayed narrower FWHMs and the effect of unbound PATP was not distinguished from other samples. Plasma-poly samples were characterised by lower background currents while the redox currents were similar to the ones observed with UV-poly. The existence of the reversible wave is an indication of the effective plasma polymerisation, regardless the significant etching effect.

To observe better the evolution of the redox reactions we calculated the CT from the area of the redox peaks for consecutive cycles (Fig. 5(d)). The CTs corresponding to oxidation and reduction (CT_ox_ and CT_red_) had different behaviours, while CT_red_ remained constant within the standard deviation, CT_ox_ that initially was three times higher decreased over the cycles until converging with CT_red_ on the 50^th^ cycle. The surface coverage corresponding to the observed charge transfer in Fig. 4(d) calculated from CT_ox_ for the e-poly samples is 2.7·10^−11^ mol/cm^2^ in cycle 1 and lowers to 1.12·10^−11^ mol/cm^2^ in cycle 50 (*S* = *CT/nF*, where *n* is 2 due to the number of electrons for each reaction in our case and *F* is the Faraday constant). The degradation of CT_ox_’s may be due to imperfect recovery of protons by the amine groups or by the degradation of the molecule (e.g. loss of aromaticity in the benzene ring that would increase the resistivity of the molecular system). In the UV and plasma-poly samples the CT_ox_’s were constant while the CT_red_’s that were initially slightly higher than the oxidation, tend to decrease with the following cycles reaching values below CT_ox_. A possible reason for this behaviour in UV and Plasma poly films is the contribution from residual e-poly occurring at the highest biases of the CV. This residual e-poly that occurs mainly in the first cycles increases the number of reversible states, which are then observed first in the following reduction peak within the same cycle. The corresponding surface coverage in UV-poly samples ranges between 1.97·10^−11^ and 1.02·10^−11^ mol/cm^2^ for cycle 1 and 50 respectively while for the UV poly sample ranges between 1.87·10^−11^ and 1.08 mol/cm^2^ for cycle 1 and 50 respectively. Plasma-poly CT’s showed similar absolute values, which is remarkable considering the decrease of the organic content due to etching. At cycle 50 the CT of all the polymerisation methods converged to similar values.

The shape of redox peaks is associated mainly with the homogeneity of the layers although factors such as neighbour interactions, SAM organisation or substrate crystallinity may increase this value^1^. To understand the impact of the polymerisation method on the reversibility, we analysed only the oxidative peaks that exhibited lower FWHM and seemed to be less affected by the unbound thiols (Fig. 5(e)). The theoretical width associated with an ideal film in the case of no interactions is 353*RT*/*nF*. (being *R* the ideal gas constant, *T* the temperature, *F* the Faraday constant and *n* the number of electrons associated with each reaction (2 in our case)). This number is *~45* mV at room temperature. Our values are much higher and the difference FWHM cannot be fit with the simple non interaction model of a single molecule. We observed a large difference between UV and e-poly samples that decreased from 41 mV on the first cycles to 26 mV at the 50^th^ cycle. Since the oxidised and reduced species of the proton exchanged reactions are not charged, and the low total surface density attributed to the dimerized molecules, the molecule to molecule interactions should not have a large effect^34^. Therefore the large FWHM may be attributed mainly to contributions from different redox reactions originated in different dimers, like the ones presented in Fig. 1(b), rather than to factors like neighbour interactions or disorder. Consequently e-poly samples would be more heterogeneous, possibly having more contributions from redox reactions, than the UV poly. We also observed the increase of the FWHM of the plasma polymerised samples. The origin can be in the activation of some extra redox couples, as we have seen that residual electropolymerisation occurred during the cycles. The FWHM of plasma and e-poly samples coincide practically after the 20^th^ cycle.

In redox active SAMs the difference between the redox peaks (E_ox_-E_red_) increases with the distance of the redox group to the electrode^1^. Also here to understand the impact of polymerisation we took into account only the difference of E_ox_ with E_red_ from the peak we attribute to bound thiols (Fig. 5(f)). The larger peak separation observed for e-poly samples with respect to UV poly can be attributed to the effect of the larger distance of the redox groups in head to tail polymerisation (associated to e-poly) and head to head dimers (dominant in UV poly). In addition the UV dimers have a double tunnelling connection to the electrode through the two thiol bonds that would increase the conductance thus decreasing the peak separation. Plasma poly samples initially have higher E_ox_-E_red_, but their values decrease until they become below the UV poly. E_ox_-E_red_ of both e-poly and plasma poly tend to decrease for consecutive cycles which would indicate that the more reversible species are the ones that have less resistance between the electrode and the redox amine. The UV-Plasma evolution is almost constant, which is consistent with the CT behaviour observed. The global picture supports the idea that head to head species are more stable than the head to tail.

**Figure 6:**
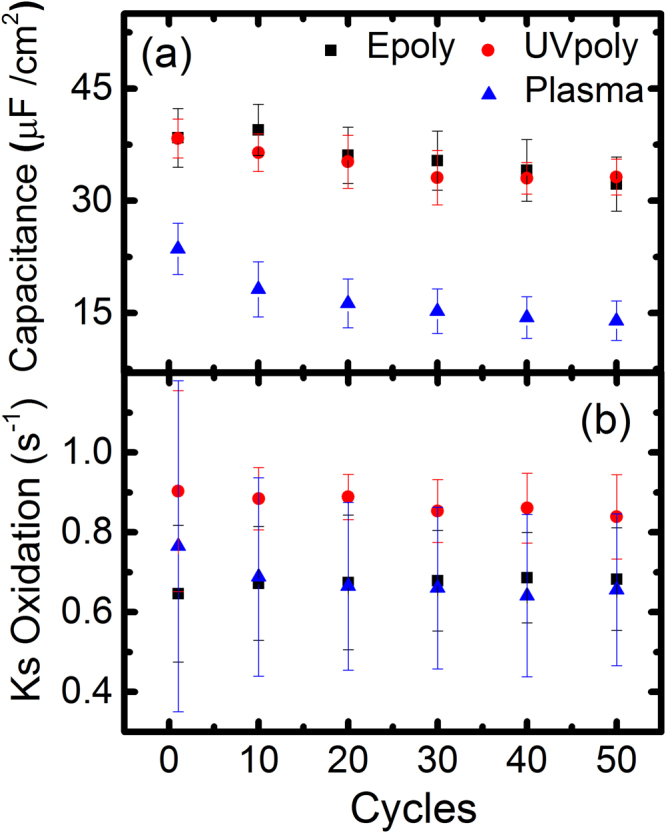
(**a**) Double layer capacitance of the polymerised films. (**b**) Electron transfer rate by the three different cycles as the function of cycles.

The double layer capacitance *C*_*dl*_ normalised to the sample surface (Fig. 6(a)) is derived from the charging current observed in the CV’s (*C*_*dl*_ = *i*_*ch*_ */ʋ*). Plasma-poly samples had lower values than the e-poly and UV-poly samples which followed similar trends. This behaviour was already noticed on the background currents described in Fig. 5(a–c). The etching occurred in the plasma treatment can explain the lower layer capacitance of the layers. As seen by XPS inelastic scattering background (Fig. 3) the thickness of the plasma-poly sample is below the PATP reference, considered as one monolayer. Therefore it is very likely that the plasma-poly sample has open pockets that become contaminated due to the high surface energy of Au, that decrease the total effective area of the layer and are reflected in a lower *C*_*dl*_. The tendency of *C*_*dl*_ to decrease in all the layers could also be interpreted as a decrease of the active area for the successive cycles due to the degradation of the conductivity of the layers.

Figure 6(b) shows the electron transfer rate (*k*_*s*_) for the oxidation potential of the different polymerised methods calculated from the maximum oxidative current divided by the charge transferred^1^. This transfer rate can only be considered as an average transfer rate of the layer since the parameters of the Gaussian functions we used to fit are not directly linked to a model from a known redox molecule^34^. However, the fact that *k*_*s*_ remains nearly constant, indicates that for e-poly and UV poly the nature of the redox species did not change significantly during the 50 cycles (since *k*_*s*_ depends on the chemical redox species). The differences and the trends are consistent with what was observed for the FWHM of the redox peaks, where plasma polymerised samples become similar to the electropolymerised after the first 10 cycles. The faster exchange rate of UV-poly samples is also consistent with the interpretation of a lower impedance of head to head dimers, due to the parallel thiol connection of the redox groups with the electrodes respect to the linear impedance in head to tail polymerisation.

## Conclusions

In this article we have used electro, UV and plasma polymerisation to induce quasi-reversible redox states. The results are interpreted in terms of polymerised dimers with reversible proton exchange reactions. In literature there is a well-established description that electropolymerisation produces head to tail dimers, while UV polymerisation can have different contributions of head to head dimers^5^. Plasma polymerisation of PATP SAMs has not been used to the date, and the chemical nature is not yet mentioned in literature. We have seen that all the polymerisation processes increase the contamination and oxidation of the layers as seen by XPS measurements, which can be due to the single thiols left by the head to tail polymerisation (observed in electropolymerisation), to the degradation of the molecules (by reactive species in plasma or UV polymerisation) or to the etching effect (mainly occurring by the effect of the plasma). The comparison of the *N1s* core spectra from the PATP reference samples and the polymerised ones provided a signature of their polymerisation observed by the splitting of the main amine peak (-*NH2*) into benzenoic diamide (*-NH-*) and diimine nitrogen (*-N* = ). The study of the *S* 2*p* core spectra showed that in electropolymerised samples the amount of thiols was bigger for electropolymerised samples, attributed to the oxidation of the single thiols. The presence of unbound thiols was also detected in some samples, which was consistent with an observation increase in the layer thickness and the shape detected by the XPS inelastic scattering background. However in the CV’s the effect of unbound thiols decreased over consecutive cycles.

Initially electropolymerisation provided the most effective technique to produce redox exchange dimers, but after 50 cycles they degraded to the same CT value achieved by other polymerisation methods. The shape of the CV curves also indicated differences in the nature of the layers. The large FWHM that we observe in all the cases is interpreted in terms of sample heterogeneity, in particular electro and plasma polymerised layers provided the higher FWHM than UV. Even if head to tail and head to head polymerisations may be predominant for electro and UV polymerisation respectively, the contribution of other species cannot be discarded. UV and plasma polymerisation initially had a much lower activation but the electronic structure of the dimers produced by these methods seemed to have advantages as they did not seem to degrade over large cycles (in other experiments not shown they were stable up to 100 cycles). The behaviour of the UV polymerised dimers were the closest to the ideal redox active SAM with lower FWHM, E_ox_-E_red_ and higher K_s._ Its structure with two thiols in parallel seems to have advantages with respect to the linear structure of the head to tail polymerisations, and these layers suffer less degradation. The characteristics of the CVs from plasma polymerised layers are more similar to electropolymerised layers in the FWHM and K_s_, but E_ox_-E_red_ was similar to the UV polymerised samples after 30 cycles, and they also exhibited similar stability.

To produce alternatives to e-poly polymerisation, an ideal redox surface should have maximum possible surface coverage possible, a fast K_s_ and maximum stability. From our experiments the method that would produce a molecule approaching this characteristics would be the UV functionalised layers, but they suffer limitations in the achievable surface coverage. Plasma polymerisation provided an interesting alternative since it produced a significant surface coverage of stable active redox couples considering the inactive areas left by the etching. In principle, this method can still be optimised and these etched areas offer the possibility of a second functionalisation thus further experiments of characterisation and optimisation of these layers could be of interest for improved applications.

## Methods

### Chemicals

4 Aminothiolphenol, Absolute Ethanol and Phosphate buffer solution (pH 7.2) were purchased from Sigma Aldrich. Millipore filtered water was used to dilute the electrolyte solution and for rinsing purposes.

### Electrode preparation

Si substrate with 50 nm of oxide layer were evaporated with Au (30 nm)/Ti (5 nm) using an e-beam evaporator and then diced into pieces of about >1 cm^2^ obtaining samples with low roughness (~2 nm). The evaporation was carried out under a vacuum less than 1e-7 mbar with an evaporation rate of 0.9 Å/s and the samples were placed at a distance of 90 cm from the source. We observed that the sample reproducibility was improved by immediately cleaning them under UV ozone chamber for 30 minutes. The cleaned electrode was then immersed in ethanol solution containing 50 mM of PATP for 24 hours.

### Electro-polymerization

We used a Faraday cage to carry on our experiments, a Pt wire as counter electrode, cleaned with sand paper and ethanol, and a calomel reference electrode. For electropolymerization the standard parameters were: scan range 0.7 to −0.2 V, scan rate –100 mV/s, cycles 1 + 3. We used 10 mM of Phosphate buffer solution as the standard electrolyte for the results presented in the article, which gave the highest CT results. However we also explored electropolymerisation in acid and basic solutions (see SI Fig. 1). All the solutions were purged with N_2_ during 5 minutes. Samples could be then studied or rinsed with ethanol and placed in Buffer solution for XPS studies.

### Photo-polymerization

Following different references^13–15^ we followed photo-polymerisation of PATP in liquid. The functionalized PATP Au electrodes were immersed in neutral phosphate buffer solution (0.1 M) for about 30 minutes, under UV light of 365 nm wavelength and power 0.9 mW/cm^2^. Also in this case we explored acidic and basic buffers obtaining also less CT than the case of neutral buffers (see SI Fig. 1).

### Plasma polymerisation

Plasma polymerisation was performed in air at atmospheric pressure with a plane-to-plane direct dielectric barrier discharge (DBD) configuration with different conditions of flux, power, and number of cycles. The DBD configuration used in this study was already described elsewhere^22^. The discharge was produced between two plane parallel high voltage electrodes (15 × 300 mm^2^ each) covered by an alumina dielectric barrier and moving stage as the grounded electrode. The gas gap between the high voltage electrodes and the substrate was maintained at 1 mm. During the deposition process, the plasma was ignited using a Corona generator from SOFTAL electronic GmbH, generating a 10 KHz sinusoidal signal. Plasma treatments were carried out using a modulated sinusoidal electrical excitation with successive ON-time (250 µs) and OFF-time (350 µs) pulses.

We explored *Ar* and *N*_2_ plasma using different cycles of our mildest possible conditions with a standard pulsed AC generator (mean power 1-2 W, power density 25–50 mW/cm^2^), voltage 0.4–0.6 KV, frequency 10 KHz, with successive 250 µs plasma ON duration and 350 µs plasma OFF duration, sample displacement rate 20 mm/s, plasma treatment length 2 cm and gas flow 20 L/min on a blanket plasma reactor. We obtained the highest charge transfer rate for the process with two runs using *Ar* plasma. As optimisation of plasma treatment is not the focus of the article, here we have presented only the results of our best plasma treatment. To reduce the variability between experimental conditions in a non-dedicated plasma reactor, after optimization of the conditions, we polymerised the batch of samples here reported on the XPS and CV analyses.

### XPS Measurements

Experiments were carried on polymerised and reference samples using two samples for each method and performing the analyses on two separated points on each of the samples. XPS measurements were carried out with a Kratos Axis Ultra DLD spectrometer using a monochromatic Al Kα radiation (primary energy E = 1486.6 eV) operating at 300 W. High resolution spectra (20 eV pass energy) were acquired for the quantification and peak fit. Electron binding energies were calibrated using the Au 4f_7/2_ transition at 84.0 eV. The quantification were obtained after the removal of a Shirley type background. The O 1 s, N 1 s, C 1 s and S 2p peak fit was performed with a symmetric Voigt function with a Lorentzian-Gaussian ratio of 30–70. The same full width at half-maximum (FWHM) for both components, a spin-orbit splitting of 1.2 eV and a branching ratio of 2 were used for the S 2p_3/2_ and S 2p_1/2_ contributions. The thickness and coverage values of the films were determined by using the Quases software [http:\quases.com] on large Au 4 f spectra acquired with 160 eV pass energy. The fits were analysed using the least number of peaks to optimise the standard deviation of the spectra.

### Electrochemistry

We extracted data from 4 samples for electropolymerisation 3 samples for UV polymerisation and 5 samples for plasma polymerisation. All the samples were evaporated with Au, functionalized with 4ATP and polymerized on the same day to avoid variability of the experimental conditions. The electrochemical experiments were carried out in a three electrode cell configuration with 4ATP functionalized Au working electrode, Pt wire counter electrode and KCl saturated Ag/AgCl reference electrode working in a Faraday cage. The electrolyte was phosphate buffer solution of 10 mM concentration. Before each experiment, the electrolyte was purged with *N*_*2*_ for about 10 minutes.

We used a Princeton applied research Versastat MC, to record cyclic voltammetry measurements of the electro and UV polymerised samples and a Solartron XM PSTAT 1 MS/s for the plasma polymerised samples.

The scan rate (ʋ) of 100 mV/s provided the best signal to noise ratio while assuring the complete polymerisation of the film (data presented in SI Fig. 2) so unless indicated other ways, all the results reported correspond to that rate. All the reported currents were normalised using the area of the samples immersed in liquid. The CVs were studied for over 50 cycles to extract the data about CT, sample homogeneity, layer capacitance and reaction rates. The results reported in Fig. 5 correspond to the same batch of samples reported on the XPS measurements.

The CV curves were fitted using the second derivative to determine the inflection points to calculate the redox peaks and determine the points at which the capacitance currents were taken.

## Electronic supplementary material

Supplementary Information
