# Supplementary material for: Influence of polymerisation on the reversibility of low-energy proton exchange reactions by Para-Aminothiolphenol
Source: Sci Rep. 2017 Nov 13;7:15401. doi: 10.1038/s41598-017-13589-5 (PMC5684359; doi:10.1038/s41598-017-13589-5)
Supplement: Supplementary file 1 — Supplementary Information [file 41598_2017_13589_MOESM1_ESM.pdf]

Influence of polymerisation on the reversibility of low-energy proton exchange reactions by Para-Aminothiolphenol

Divya Balakrishnan[a,b], Guillaume Lamblin[a], Jean Sebastien Thomann[a], Jerome Guillot[a], David Duday[a], Albert van den Berg[b], Wouter Olthuis[b], César Pascual-García[a]

[a]Luxembourg Institute of Science and Technology (LIST), 41 Rue du Brill, L-4422 Belvaux, Luxembourg
[b]MESA+ institute, University of Twente, Drienerlolaan 5, 7522 NB Enschede, Netherlands

Suplementary information

UV wavelength – 365nm, Power = 0.88mW/cm2, scan rate – 0.1V/s,

PBS buffer – pH 7.4

Sample A

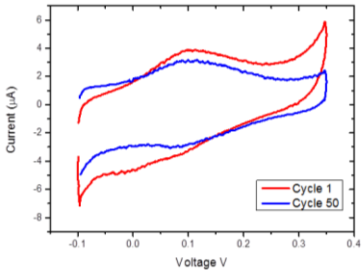

Time – 15mins

Sample B

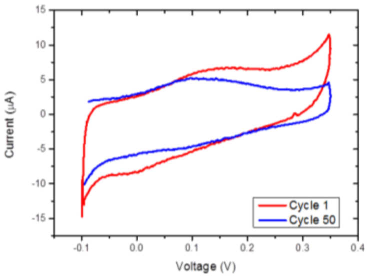

Time – 30mins

Surface Coverage (mol/cm2)

4.04E-11 / 3.55E-11 **1.57E-11 / 2.73E-11**

PBS buffer – pH 5.2

Sample C

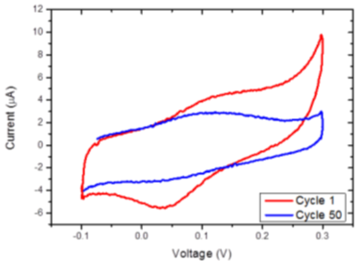

Time – 15mins

Sample D

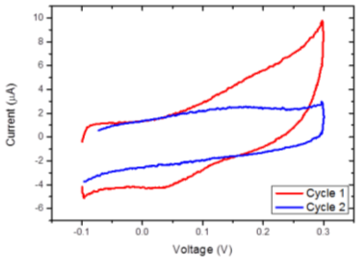

Time – 30mins

Surface Coverage (mol/cm2)

8.47E-11 / 2.68E-11 **Flat no Redox peaks**

PBS buffer – pH 9.0

Sample E

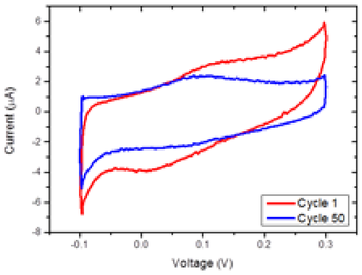

Time – 15mins

Sample F

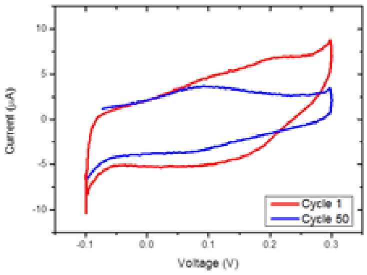

Time – 30mins

Surface Coverage (mol/cm2)

7.11E-12 / 1.35E-11 **1.55E-11 / 1.92E-11**

SI Figure 1 UV polymerisation of different samples at neutral (samples A, B) acid (samples C, D) and basic (samples E, F) buffer conditions. Below the surface coverage determine from Oxidation reactons is expressed in red and blue for the first and 50th cycle respectively.

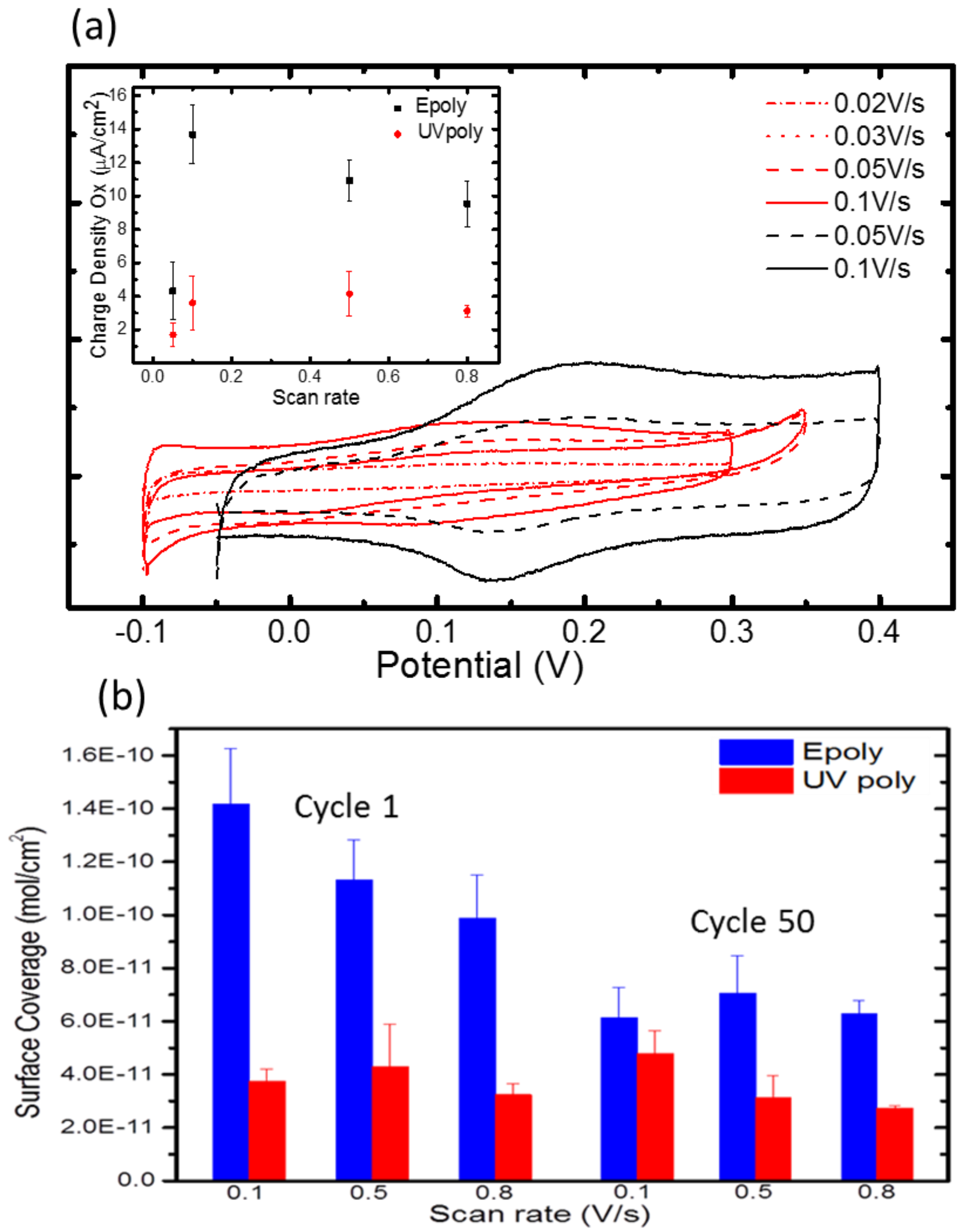

SI Figure 2 *(a)* cyclic voltammetry measurements carried on both Electro and UV polymerised samples for different scan rates ranging from 0.02V/s to 0.1V/s. the inset graph shows the oxidation peak charge density against the scan rate for Electro and UV polymerised samples. (b) Surface coverage vs scan rate derived from oxidation reactions of different e-poly and UV poly samples (cycle 1 is first cycle after electropolymerisation).

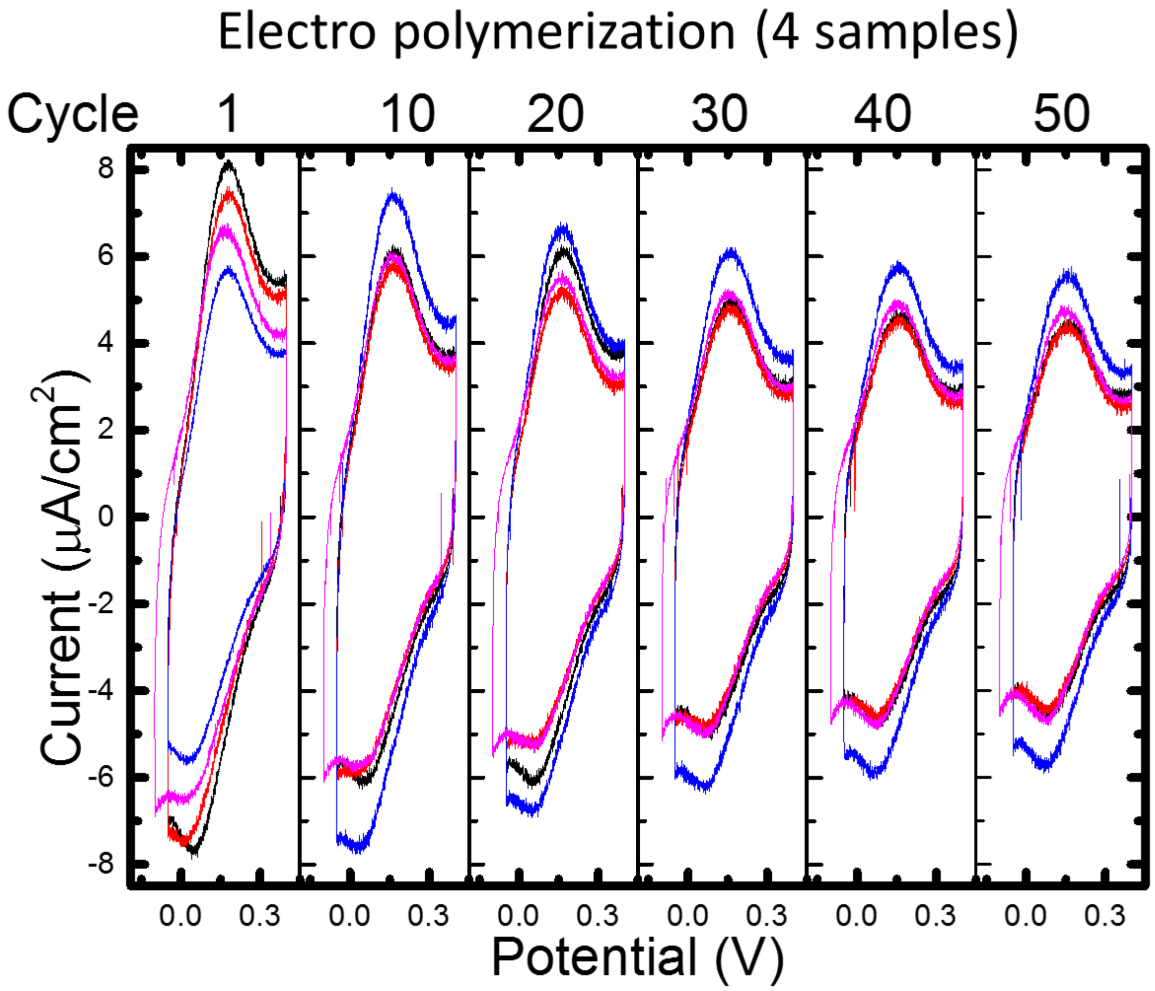

SI Figure 3 Raw data corresponding to the different electro-polymerised samples used to calculate the data in fig 5 a-f and 6 (cycle 1 is first cycle after electropolymerisation)

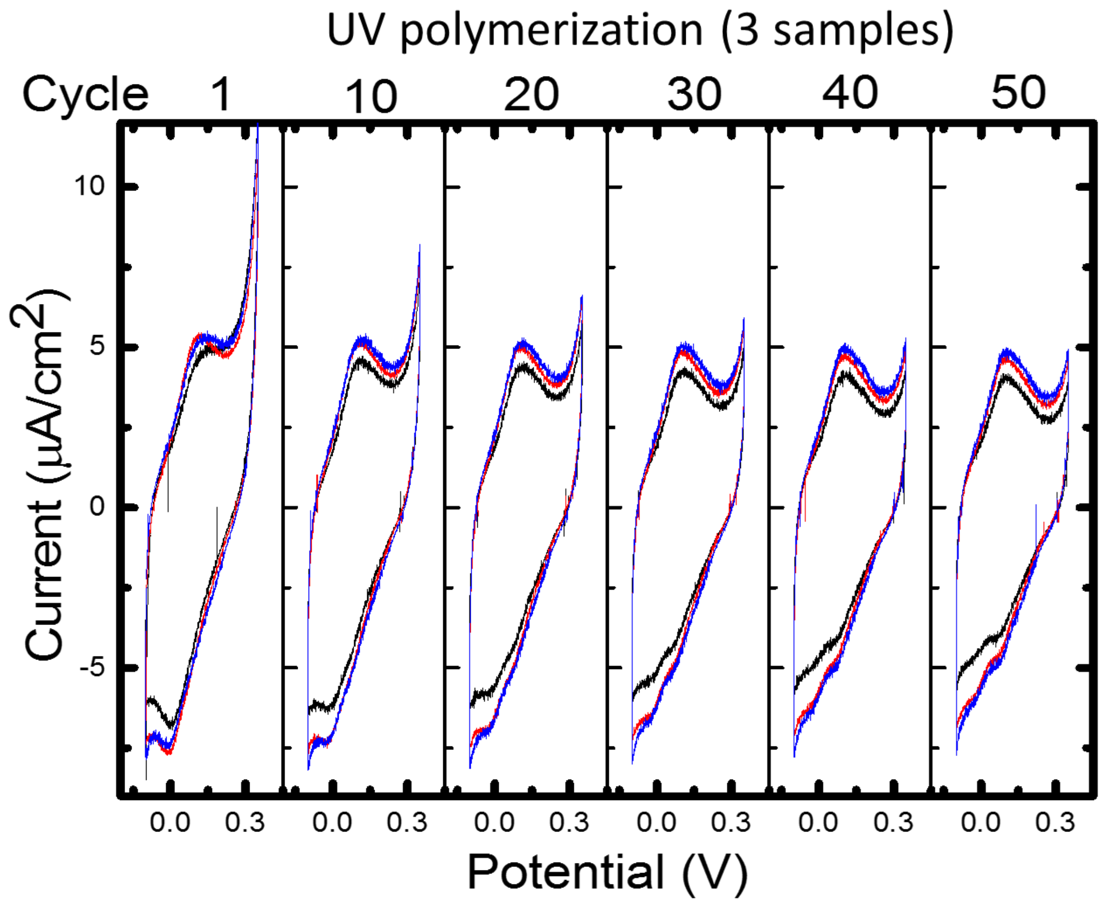

SI Figure 4 Raw data corresponding to the different UV polymerised samples used to calculate the data in fig 5 a-f and 6

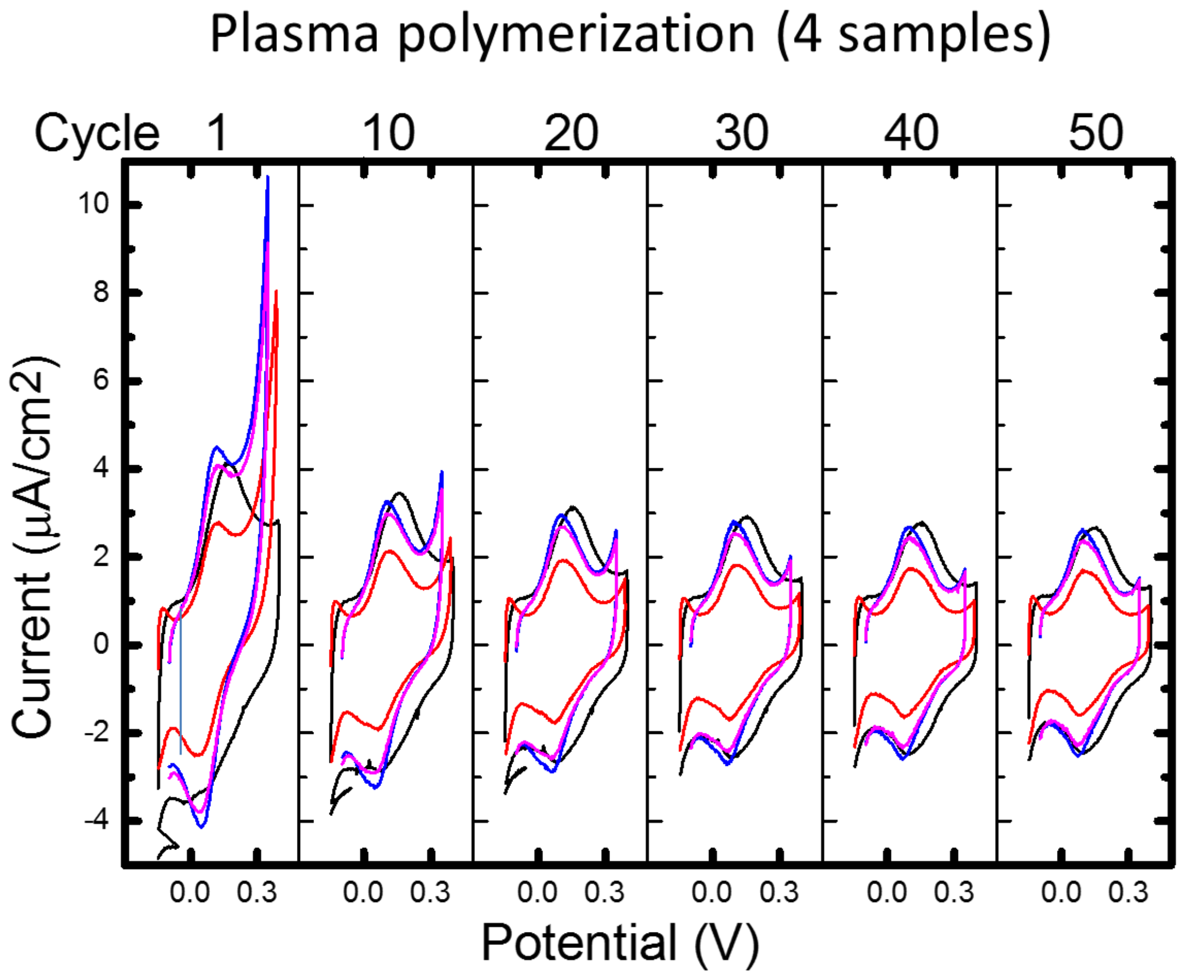

SI Figure 5 Raw data corresponding to the different Plasma polymerised samples used to calculate the data in fig 5 a-f and 6
